# 531 Utilization of a Nurse Care Coordinator to Streamline the Outpatient Experience

**DOI:** 10.1093/jbcr/irad045.128

**Published:** 2023-05-15

**Authors:** Joya Neal, Tiffany Hockenberry, Karen Richey, Kevin Foster

**Affiliations:** Arizona Burn Center @ Valleywise Health Medical Center, Gilbert, Arizona; AZ Burn Center at Valleywise Health, Phoenix, Arizona; Arizona Burn Center Valleywise Health, Phoenix, Arizona; AZ Burn Center at Valleywise Health, Phoenix, Arizona; Arizona Burn Center @ Valleywise Health Medical Center, Gilbert, Arizona; AZ Burn Center at Valleywise Health, Phoenix, Arizona; Arizona Burn Center Valleywise Health, Phoenix, Arizona; AZ Burn Center at Valleywise Health, Phoenix, Arizona; Arizona Burn Center @ Valleywise Health Medical Center, Gilbert, Arizona; AZ Burn Center at Valleywise Health, Phoenix, Arizona; Arizona Burn Center Valleywise Health, Phoenix, Arizona; AZ Burn Center at Valleywise Health, Phoenix, Arizona; Arizona Burn Center @ Valleywise Health Medical Center, Gilbert, Arizona; AZ Burn Center at Valleywise Health, Phoenix, Arizona; Arizona Burn Center Valleywise Health, Phoenix, Arizona; AZ Burn Center at Valleywise Health, Phoenix, Arizona

## Abstract

**Introduction:**

Often being discharged from the hospital can be a frightening experience for burn patients who have complex and multifaceted outpatient needs. Our center found that while clinical staff were able to manage the immediate needs of the patient, they were unable to provide the level of support needed for patients to reach their full rehabilitation potential. In response to these findings, burn leadership developed the outpatient Nurse Care Coordinator (NCC) role, to ease the transition to the outpatient setting and enhance support. The purpose of this project was to evaluate the utilization and efficacy of the new NCC role.

**Methods:**

This was a retrospective 1.5-year review of activities performed by the NCC directly relating to patient services. Five domains were chosen for monthly tracking, individual number of patients assisted, external referrals, workman’s compensation patients, compression garment orders and PTSD screenings. Basic descriptive statistics were calculated.

**Results:**

This position has been in place for 2 years. The mean number of patients followed by the NCC was 592/month (204-723), average external referrals 43/month (11-84), average workman’s compensation patients 43.4/month (30-59), total compression garments ordered 54.8/month (21-106) and average number of PTSD screening 47.4/month (1-129). External referrals encompassed home health services, outpatient therapies, and multiple providers resulting in 1-15 interactions/patient to establish ordered services. Workman’s compensation patients required coordination with insurance, care managers, employers and adjusters including required documentation. Compression garments were tracked for progress on fulfilment, arranging assistance for financial coverage, required documentation, and coordinating measurements and delivery with patients amongst multiple providers. For patients screening positive for PTSD, the NCC provided resources and multiple options for PTSD/trauma counselors for the patient’s mental wellbeing.

**Conclusions:**

The dedicated outpatient Nurse Care Coordinator is uniquely positioned to facilitate and optimize the care of patients in the outpatient arena. While data is not available for direct comparison prior to institution of this role, anecdotally, vendors, patients, clinic staff and providers report improved continuity and compliance with recommendations. We continue to refine and enhance the responsibilities of the NCC to better serve our patient population.

**Applicability of Research to Practice:**

The Nurse Care Coordinator is essential in helping the survivor with complex needs, successfully navigate the complex and multidimensional outpatient setting.

